# A Novel Aerial-Aquatic Unmanned Vehicle Using Flapping Wings for Underwater Propulsion

**DOI:** 10.3390/biomimetics9100581

**Published:** 2024-09-25

**Authors:** Jiacheng He, Yingjie Zhang, Junjun Feng, Shisheng Li, Yiheng Yuan, Pinghui Wang, Song Han

**Affiliations:** School of Innovation and Entrepreneurship, Southern University of Science and Technology, Shenzhen 518055, China; hejc@mail.sustech.edu.cn (J.H.); yingjie_zz@163.com (Y.Z.); fjj2661@126.com (J.F.); 12032546@mail.sustech.edu.cn (S.L.); 12233169@mail.sustech.edu.cn (Y.Y.); wangph@sustech.edu.cn (P.W.)

**Keywords:** aerial-aquatic unmanned vehicle, underwater flapping wing propulsion, diagonal motion trajectory improvement, underwater propulsion speed

## Abstract

Aerial-aquatic unmanned vehicles are a combination of unmanned aerial vehicles and unmanned submersibles, capable of conducting patrols in both the air and underwater domains. This article introduces a novel aerial-aquatic unmanned vehicle that integrates fixed-wing configuration and flapping-wing configuration. In order to improve the low efficiency of the classic diagonal motion trajectory, this paper proposed an improved diagonal motion trajectory based on joint optimization of the stroke angle and angle of attack curve. The proposed method has been verified through simulations and experiments. A prototype was developed and experiments were completed, both indoors and outdoors, wherein the system’s transmedium transition capability and flapping propulsion performance were comprehensively validated. Additionally, utilizing flapping propulsion, an average underwater propulsion speed of 0.92 m/s was achieved.

## 1. Introduction

Aerial-aquatic unmanned vehicles possess the capability of underwater locomotion, water egress, flight, and water ingress. This transmedium aircraft combines the concealment characteristics of underwater vehicles with the high-speed capabilities of unmanned aerial vehicles, significantly broadening the application scope for unmanned aerial vehicles. It finds diverse applications in marine environmental detection and national defense security, owing to its unique capabilities [1,2,3].

Presently, mature aerial-aquatic unmanned vehicles are primarily designed based on fixed-wing or rotary-wing drones to enable transmedium flight. For instance, fixed-wing drones adopt variant wing designs [4], while rotary-wing drones incorporate multirotor propulsion devices for water egress and ingress [5]. The development of aerial-aquatic unmanned vehicles with fixed-wing configurations has reached a considerable level. William Stewart et al. from the North Carolina State University developed the EagleRay series of aerial-aquatic, fixed-wing unmanned aerial vehicles [6,7]. They have a single motor and propeller combination for propulsion in aerial and underwater domains, and the maximum underwater locomotion speed reaches 0.89 m/s. Friedrich et al. from ETH Zurich designed a foldable wing amphibious fixed-wing unmanned aerial vehicle named Dipper [4,8]. Dipper utilizes fixed-wing flight in the air and folds its wings underwater, relying on a single motor and dual propellers to propel it, and the maximum underwater speed is 3 m/s. Xingbang Yang et al. from Beihang University proposed a hybrid aerial-underwater vehicle with foldable wings. They designed a balloon upright system that allows the vehicle to stand upright within a limited transition time, enabling the coaxial counter-rotating propellers to be positioned away from the water surface, thereby providing sufficient thrust to pull the vehicle out of the water [9,10]. Zhaoyu Wei et al. from Shanghai Jiao Tong University proposed a delta-wing layout, fixed-wing unmanned aerial-aquatic vehicle. Unlike other aerial-aquatic unmanned vehicles, it has relatively large net buoyancy and can stably float on the water surface, achieving submersion through the generation of negative lift by the wings [11]. However, the current aerial-aquatic unmanned vehicles based on fixed-wing configurations have problems such as high noise and propulsion system compatibility between air and water. Due to the significant difference in the density of air and water, the propellers used in the air and underwater are not compatible. Meanwhile, propeller propulsion is unfriendly to aquatic life and could create much disturbance to the underwater ecosystem.

A pivotal aspect in the research and development of amphibious drones lies in underwater propulsion technology. The propulsion technology of underwater robots provides a reference for amphibious drones. Currently, in addition to propeller propulsion, the propulsion technology of underwater robots mainly includes jet propulsion, glide propulsion, fin-based propulsion, and so on. Jet propulsion only shows a good ability for rapid flee dangers, which cannot meet the requirements of complex underwater operations [12]. Chen et al. devised a jet propulsion device constructed with 80% soft materials that has the capability to modify its cavity volume, enabling absorption and ejection of fluid media for robot movement [13]. Glide propulsion enables long-endurance underwater navigation, but it has poor maneuverability owing to insufficient drive force [14], and their routes are prone to deviations. Sun et al. explored the efficiency of “Black Pearl” wave glider flapping hydrofoils under different spring stiffness coefficients and limited pitch angles, with the maximum average speed reaching 0.372 m/s [15].

From a bionics perspective, the swimming principles of aquatic creatures can provide more inspiration for underwater propulsion technology. Researchers have shifted their focus toward the exploration of fin-based propulsion techniques, whose propulsion modes can be broadly categorized as body/caudal fin (BCF) propulsion, median/paired fin (MPF) propulsion, or their hybrid modes [16,17]. BCF locomotion can produce relatively larger thrust in still-water environments, which is suitable for long-time, high-speed cruising [17]. Katzschmann et al. created a soft robotic fish named SoFi, which is actuated by a hydraulic pump and can conduct approximately 40 min of continuous observation at a maximum depth of 18 m [18]. J. Zhu et al. developed a 255-millimeter-long Tunabot robot that mimics a tuna, using a single mechanical joint to connect rigid fins. It can achieve a maximum speed of 4 body lengths per second [19]. The MPF propulsion mode has better performance in terms of mobility, stability, and concealment of swimming process [20]. Jason D. Geder et al. from the U.S. Naval Laboratory designed a bionic flapping-wing submarine called ’Flimmer-s’ [21,22,23]. Its pectoral fin employs a four-fin drive scheme, where four carbon-fiber fin-bars, with a specific phase difference, propel the nylon wing membrane, simulating the movement of natural pectoral fins. Li et al. designed the electronic fish with silicone thin film as flapping pectoral fins, which was driven solely by a soft electroactive structure made of dielectric elastomer and ionically conductive hydrogel, and it can swim at a speed of 6.4 cm/s [24]. Pan Guang et al. from Northwestern Polytechnical University have created a soft-body submersible mimicking the sliding and flapping motions of a manta ray, employing a flexible skin design around a pectoral fin skeleton in their vehicle design [25]. Soheil Arastehfar et al. developed an underwater robot named MantaDroid, which mimics a manta ray. Its flexible fins are made of polyvinyl chloride sheets. Each flexible fin is powered by a Hitec HS-5646WP servomotor, providing relatively high swimming speed and long endurance [26]. K. H. Low et al. designed a robot named RoMan-III that mimics the swimming motion of a real manta ray. Its fin design includes three independently driven servo motors, each motor driving three parallel-connected fin skeletons. The fin skeletons are made of Teflon material to increase flexibility. RoMan-III is more compact in size while maintaining swimming speed [27]. Fin-based propulsion is safer for aquatic life, while being quieter and having better concealment performance. Hence, the propulsion method using flapping pectoral fins provides a new solution for underwater propulsion in aerial-aquatic unmanned vehicles.

To explore a novel underwater propulsion method for aerial-aquatic unmanned vehicles, we propose a novel unmanned vehicle design that combines fixed-wing and flapping-wing configurations. In this design, the inner wing section is configured as a fixed-wing, while the outer wing section employs servos to actuate carbon-fiber fin rays covered with silicone skin to act as a flapping wing. This configuration is capable of both fixed-mode aerial flight and underwater flapping-wing propulsion. To enhance the propulsion efficiency of the diagonal motion trajectory, we proposed an improved diagonal motion trajectory based on the joint optimization of the stroke angle and angle of attack curve. In Section 2, we employ numerical simulations and water tank experiments to investigate various flapping trajectories and validate the effectiveness of the proposed trajectory. In Section 3, we design and manufactured a prototype and conduct underwater cruise speed tests and transmedium transition capability tests outdoors. Section 4 concludes by summarizing our works and outlining future research directions.

## 2. Kinematic Analysis and Hydrodynamic Analysis

### 2.1. Classical Motion Trajectory

The intricate flapping motions observed in birds or manta rays present challenges for achieving complete replication using flapping foils. To simplify this complexity, it is common to reduce the flapping motion to either a symmetric motion trajectory with two degrees of freedom (DOF) or a diagonal motion trajectory with three DOF. The symmetric motion trajectory exhibits higher flapping efficiency, while the diagonal motion trajectory generates greater thrust, albeit at lower efficiency [28].

**Figure 1:**
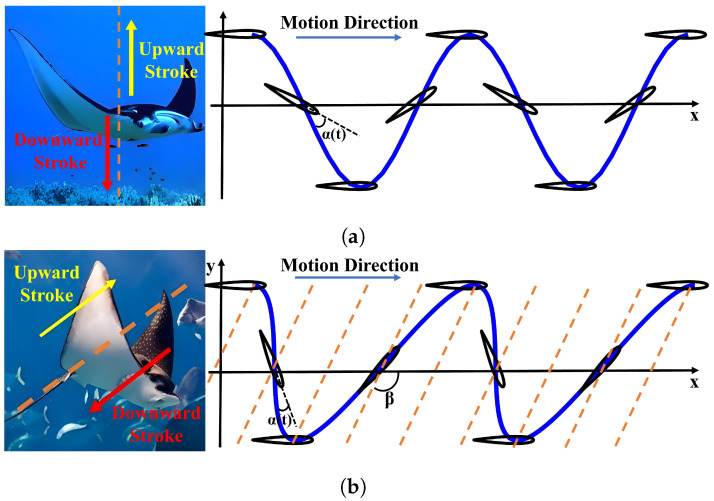
The classical underwater motion trajectory. (**a**) Classical symmetric motion trajectory. (**b**) Classical diagonal motion trajectory.

The expressions of the classical motion trajectory is shown in Table 1. The 2-DOF flapping motion typically involves the up-and-down flapping in the vertical forward direction and pitching rotation around the wing axis, resulting in a symmetric motion trajectory, as illustrated in Figure 1a. The expressions of the motion vertical direction (y direction) and parallel to the forward direction (x direction) and the change of the angle of attack (α) are given by Equations (1), (2), and (4), respectively [28]. In the downward stroke of the symmetric motion trajectory, the flapping wing motion generates a positive angle of attack, while in the upward stroke it generates a negative angle of attack. By controlling the change of the angle of attack of the upward and downward strokes, a forward thrust can always be generated by the flapping wings.

In these equations, *h* is the flapping amplitude, *f* is the flapping frequency, *U* is the flow speed, and $\alpha_{max}$ is the amplitude of the angle of attack.

Compared with the 2-DOF flapping motion, the 3-DOF flapping motion, i.e., diagonal motion trajectory, increases the DOF along the forward direction, which is controlled by the stroke angle (β). When the vehicle moves forward at constant speed *U*, it is observed that the foil translates back and forth relative to a straight line in the direction of motion. This line (the orange line in Figure 1b) is called the stroke line and is defined by the angle β relative to the horizontal plane [28,29,30]. Figure 1b shows the schematic of diagonal motion trajectory. The equation of motion for the underwater diagonal trajectory is represented by Equations (1), (3), and (5), which describe the motion in the y direction, x direction, and the change of the α, respectively [28].

The Strouhal number (St) is a fundamental parameters in hydrodynamics, which is precisely defined as follows [31]:

(6) $$St=\frac{2fh}{U}$$

In this paper, propulsion efficiency is an important evaluation criterion for different flapping motion trajectories. During the numerical simulation and experimental phases, we evaluate the propulsion efficiency of different flapping motion trajectories. When the flapping wing is in a steady state, the average work of thrust in a cycle is defined as output power, and the ratio of output power to input power is the propulsion efficiency [28]:

(7) $$P_{0}=\frac{1}{T}\int_{0}^{T}F_{x}\left(t\right)Udt$$

(8) $$P_{1}=\frac{1}{T}\int_{0}^{T}\left[F_{x}\left(t\right)\dot{x}\left(t\right)+F_{y}\left(t\right)\dot{y}\left(t\right)+M\left(t\right)\dot{\theta }\left(t\right)\right]dt$$

(9) $$\eta =\frac{P_{0}}{P_{1}}$$

where $P_{0}$ is the output power (W), $P_{1}$ is the input power (W), η is propulsion efficiency (unitless), *T* is the flapping period (s), $F_{x}$ is the thrust (N), $F_{y}$ is the vertical force (N), *M* is the pitching moment (N·m), $\dot{x}\left(t\right)$ is the horizontal moving speed (m/s), $\dot{y}\left(t\right)$ is vertical moving speed (m/s), and $\dot{\theta }\left(t\right)$ is the twisting angular speed (rad/s).

### 2.2. Numerical Simulation and Trajectory Improvement

#### 2.2.1. Numerical Simulation Method

The dynamic mesh functionality of ANSYS FLUENT was used to implement different flapping motion trajectories in order to obtain the thrust, vertical force, and pitching moment during the flapping motion, and thereby investigate the characteristics of various flapping motion trajectories. We developed a user-defined function (UDF) to impart specific motion modes to the flapping wing. The computational domain is 40*c* × 20*c*, where *c* represents the length of the flapping wing chord. In this study, we used an NACA 0012 airfoil (Data from NASA’s 2D NACA 0012 airfoil validation case, USA, see https://turbmodels.larc.nasa.gov/naca0012_val.html, accessed on 24 September 2024) with a chord length of 55 mm. The inlet boundary was set as a velocity inlet, while the outlet boundary was defined as a pressure outlet, and non-slip wall conditions were applied to the wing boundary. A second-order upwind scheme was used for the spatial discretization and the Coupled algorithm was used for the pressure–velocity coupling. Unstructured grids with sliding and layering dynamic mesh methods were used to handle the mesh.

#### 2.2.2. Symmetric Motion Trajectory Numerical Simulation

The hydrodynamic characteristics of flapping wings in symmetric motion trajectory are influenced by the amplitude of angle of attack ($\alpha_{max}$), flapping frequency (*f*), flapping amplitude (*h*), and incoming flow speed (*U*). The effect of these variables was explored respectively. The variables *f*, *h*, and *U* are associated with the Strouhal number. Therefore, the following variable control methods were employed when investigating the influence of these variables. Specifically, *f* or *h* was varied while *U* remained constant to investigate the Strouhal number’s impact on hydrodynamic characteristics. Additionally, both *f* and *h* were modified simultaneously, while *U* was maintained at a constant value, to evaluate the effects of varying *f* on hydrodynamic characteristics while maintaining a constant Strouhal number. In the subsequent text, $C_{x}$ denotes the thrust coefficient and $C_{y}$ denotes the vertical force coefficient.

**Figure 2:**
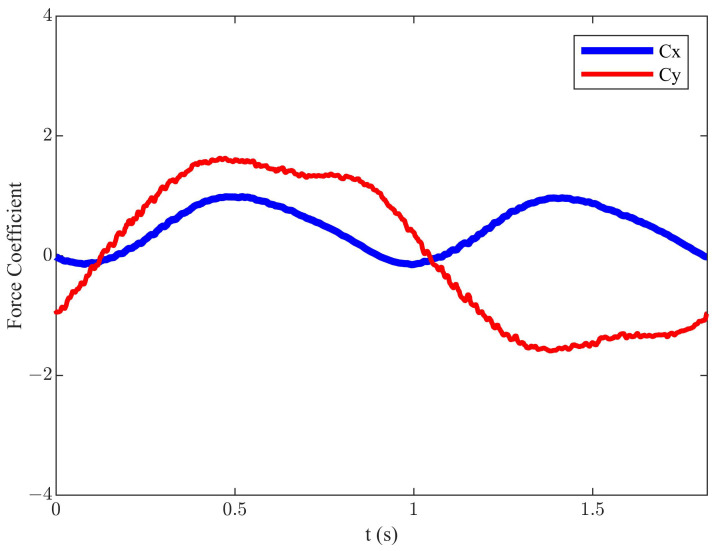
The instantaneous force coefficients of the symmetric motion trajectory.

For the symmetric motion trajectory, the thrust demonstrates an initial increase followed by a subsequent decrease as the $\alpha_{max}$ increases, and it gradually increases with an increase in flapping frequency. Following numerous simulations conducted under different $\alpha_{max}$, *f*, *h*, and *U*, as mentioned above, when $\alpha_{max}$ = 17°, *h* = 0.055 m, *f* = 0.55 Hz, and *U* = 0.2 m/s (corresponding St = 0.3025), the maximum efficiency of the symmetric motion trajectory was 57.16%, the corresponding mean $C_{x}$ was 0.45, and mean $C_{y}$ was 0.01. The simulation results depict the instantaneous variation of hydrodynamic characteristics, as illustrated in Figure 2.

#### 2.2.3. Diagonal Motion Trajectory Numerical Simulation and Improvement

**Figure 3:**
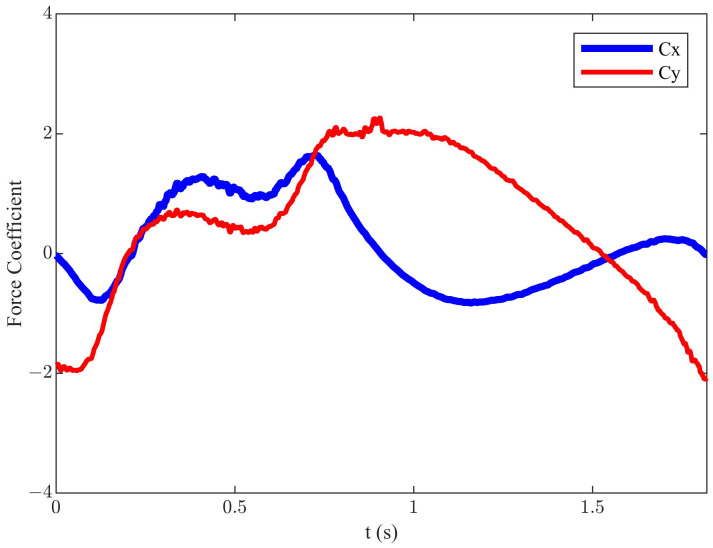
The instantaneous force coefficients of the diagonal motion trajectory.

For diagonal motion trajectory, we conducted simulations to investigate the influence of $\alpha_{max}$, *f*, *h*, *U*, and β on propulsion performance and efficiency. Under the conditions of *h* = 0.055 m, *f* = 0.55 Hz, *U* = 0.2 m/s, $\alpha_{max}$ = 25°, and β = 135° (corresponding St = 0.3025), the efficiency achieved was 24.3%, the corresponding mean $C_{x}$ was 0.12, and mean $C_{y}$ was 0.40. The simulation result of the instantaneous variation of hydrodynamic characteristics is illustrated in Figure 3.

Compared to the symmetric motion trajectory, the efficiency of the diagonal motion trajectory is lower, but it generates greater thrust. By observing Figure 3, it is found that, although the diagonal motion trajectory generates greater thrust, it also produces significant drag and vertical force throughout the cycle. This is the reason for the lower mean $C_{x}$ and efficiency during the cycle. To enhance propulsion efficiency, this paper proposed an improved diagonal motion trajectory, which was based on the joint optimization of the stroke angle and angle of attack curve. The equations of motion in the y and x directions are the same as Equations (1) and (3), and the change of the α is given as follows:

(10) $$\alpha \left(t\right)=\left\{\begin{array}{cc} \left(\alpha_{max}-\alpha_{0}\right)\times {sin}^{2}\;\left(8\pi ft/3\right)+\alpha_{0} & 0<t\leq \frac{3T}{16}\\ \alpha_{max} & \frac{3T}{16}<t\leq \frac{T}{4}\\ \left(\alpha_{max}+15\right)\times {sin}^{2}\;\left(2\pi f\left(t-T/2\right)\right)-15 & \frac{T}{4}<t\leq \frac{T}{2}\\ \left(\alpha_{0}+15\right)\times {sin}^{2}\;\left(\pi f\left(t-T/2\right)\right)-15 & \frac{T}{2}<t\leq T \end{array}\right.$$

where $\alpha_{0}$ is the preset angle of attack.

**Figure 4:**
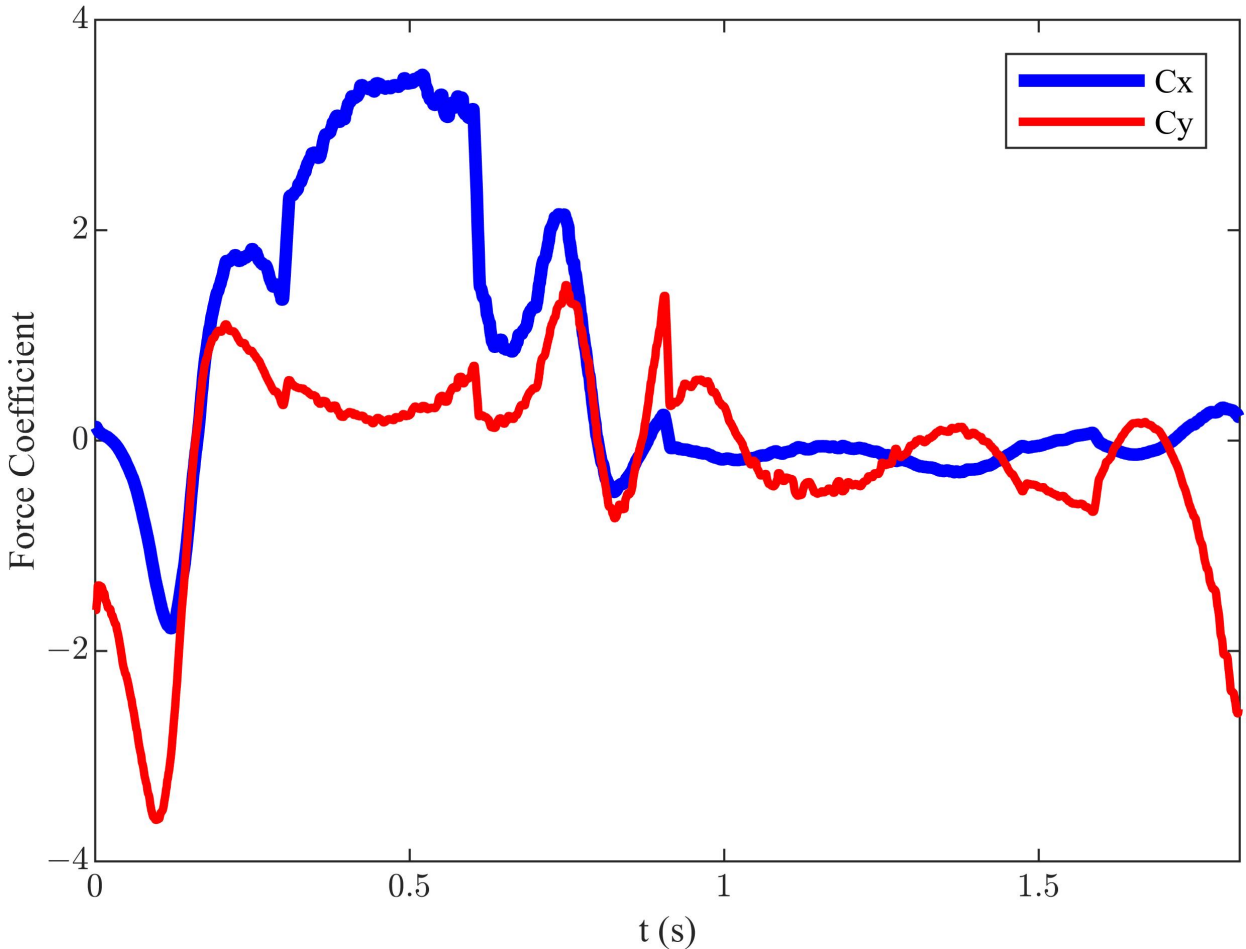
The instantaneous force coefficients of the improved diagonal motion trajectory.

Under the conditions of *h* = 0.055 m, *f* = 0.55 Hz, *U* = 0.2 m/s, β = 144°, $\alpha_{0}$ = 5°, and $\alpha_{max}$ = 40° (corresponding St = 0.3025), an efficiency of 34.4% was achieved. This represents a 10.1% increase compared to the classical diagonal motion trajectory. The instantaneous force coefficients for this state are shown in Figure 4. Compared to the classical diagonal motion trajectory, the corresponding mean $C_{x}$ had significantly increased to 0.65, and mean $C_{y}$ was decreased to −0.15.

### 2.3. Water Tank Experimental Verification

The water tank experiments were based on the improved diagonal motion trajectory obtained from simulations to further explore the propulsion performance of the flexible flapping wing prototype.

#### 2.3.1. Experimental Apparatus

**Figure 5:**
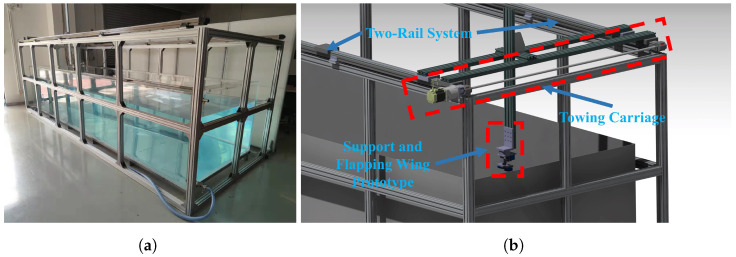
The experimental towing tank schematic. (**a**) Towing tank. (**b**) Towing carriage schematic.

The experiments were conducted in a towing tank (Shenzhen Yinfei Electronic Technology Co., Ltd., Shenzhen, China) with dimensions of 5 m (length) × 1.5 m (width) × 1 m (height), as shown in Figure 5. The water tank was equipped with a towing carriage, operating at speeds ranging from 0.03 m/s to 1.5 m/s, capable of movement along a two-rail system, and the experimental prototype rests on the support that moves forward through the tank at a constant velocity, as illustrated in Figure 5b. The force measurement system uses ATI’s Gamma 6-DOF force and torque sensors (ATI Industrial Automation, Inc., Apex, NC, USA), and its sampling frequency is 7000 Hz.

**Figure 6:**
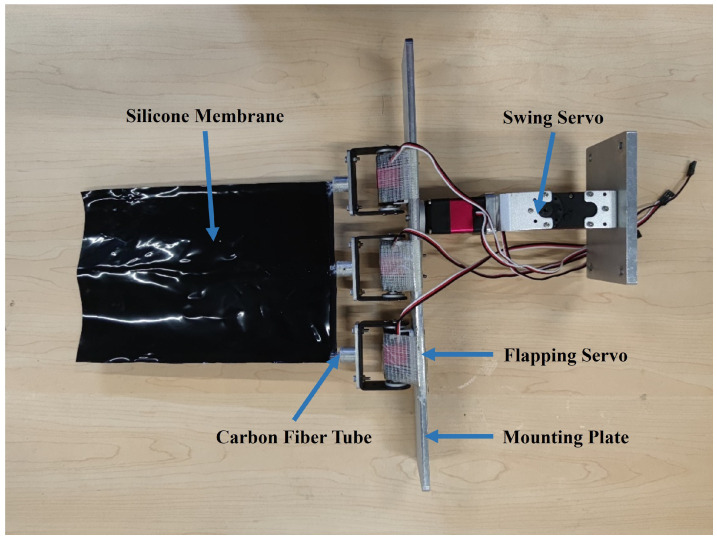
The flexible flapping wing prototype.

To achieve symmetric and diagonal motion trajectory flapping, this paper designed a flexible flapping wing prototype for underwater testing, as shown in Figure 6. Taking into consideration the need to maintain the wing shape and the complexity of the system, as well as weight limitations, the flapping wing is controlled through the different deflections of three fin rays, and a 1 mm silicone membrane covers the fin rays for the skin. Each fin ray is driven and controlled by a servo motor (Hiwonder Co., Ltd., Shenzhen, China). In addition, an extra servo motor is employed to control the stroke angle. The clamping plate of the transducer and the connecting plate of the linear module were manufactured with aluminum alloy. STM32 (STMicroelectronics NV, Plan-les-Ouates, Switzerland) was selected as the core chip of the prototype, and it sends out a PWM signal to achieve angular control of the servo motor.

To achieve symmetric motion trajectory flapping, the swing servo remains fixed while the flapping servos are controlled by sending PWM signals to achieve the upward and downward flapping of the wing. When there is a phase difference among the three flapping servos, it simulates flapping wing flexural deformation. To achieve diagonal motion trajectory flapping, the swing servo is additionally controlled to enable the forward and backward swinging motion of the wing on top of the aforementioned setup.

**Figure 7:**
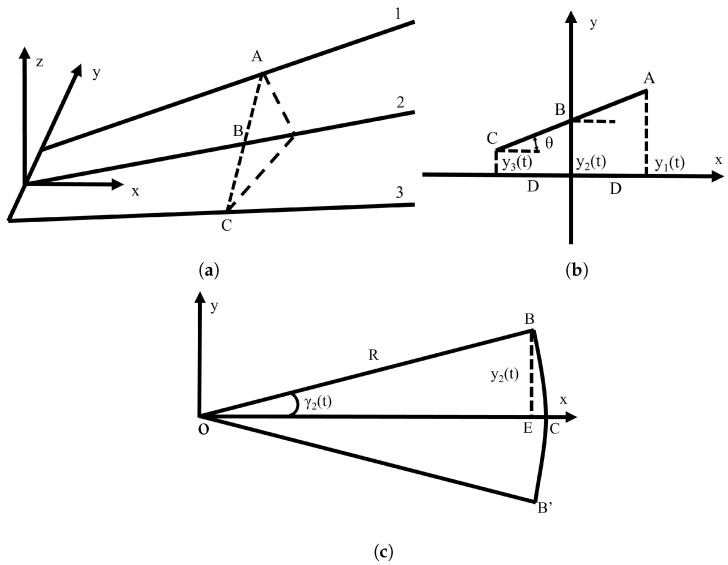
Schematic of fin rays’ motion. (**a**) The position of fin rays at a certain time. (**b**) Relative position of fin rays at 0.7 times the span of the fin ray. (**c**) Schematic of motion of fin ray 2.

The following shows how the fin changes its shape based on the servos’ movement. Figure 7a shows the position of three fin rays at a certain moment, and Figure 7b shows the relative position of three fin rays at 0.7 times the span of the fin ray. Figure 7c is the schematic of the motion of fin ray 2, where *R* is the distance between the rotating axis and 0.7 times the span of the fin ray, o is the location of the rotating axis of the servo, B is the upper limit of 0.7 times the span of the fin ray, and γ is the rotation angle of the fin rays. Fin ray 2 is the rotating center of the twist motion; the motion equation of fin ray 2 is:

(11) $$y_{2}\left(t\right)=hcos\;\left(2\pi ft\right)$$

According to Figure 7b, the motion equations of fin ray 1 and fin ray 3 are as follows:

(12) $$y_{1}\left(t\right)=y_{2}\left(t\right)+D\times tan\;\theta$$

(13) $$y_{3}\left(t\right)=y_{2}\left(t\right)-D\times tan\;\theta$$

where *D* is the distance between two adjacent fin rays and θ is the twist angle of the flapping wing.

As the rotation angle of fin rays in this paper is small, it is reasonable to substitute BC with BE; the rotating angle can be expressed as follows:

(14) $$\gamma_{2}\left(t\right)=arcsin\;\left(\frac{y_{2}\left(t\right)}{R}\right)=arcsin\;\left(\frac{hcos\;(2\pi ft)}{R}\right)$$

(15) $$\gamma_{1}\left(t\right)=arcsin\;\left(\frac{y_{1}\left(t\right)}{R}\right)=arcsin\;\left(\frac{hcos\;(2\pi ft)+D\times tan\;\theta }{R}\right)$$

(16) $$\gamma_{3}\left(t\right)=arcsin\;\left(\frac{y_{3}\left(t\right)}{R}\right)=arcsin\;\left(\frac{hcos\;(2\pi ft)-D\times tan\;\theta }{R}\right)$$

In the same way, we can obtain the equation for the swing servo:

(17) $$\gamma_{4}\left(t\right)=arcsin\;\left(\frac{x(t)}{R^{\prime }}\right)=arcsin\;\left(\frac{hcos\;(2\pi ft)}{R^{\prime }tan\;\beta }\right)$$

where $R^{\prime }$ is the distance between the rotating center of the swing servo and 0.7 times the span of the fin ray.

Before the experiments, preliminary tests were conducted to determine the maximum achievable rotational angular velocity and the twist angle of the flexible flapping prototype. The results indicated that the maximum achievable rotational angular velocity was 76°/s, and meanwhile, the maximum twist angle could reach 43°, which is essential for subsequent experiments to adhere to these physical constraints.

#### 2.3.2. Experiment Results

**Figure 8:**
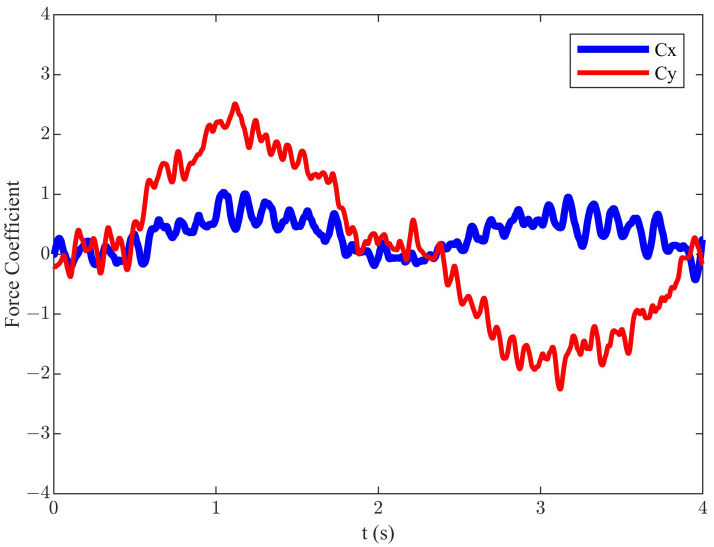
The experiment results of the instantaneous force coefficients of the symmetric motion trajectory.

Through multiple experiments on the flexible flapping wing under different operating conditions, it was ultimately found that the highest propulsion efficiency of the symmetric motion trajectory was achieved at *h* = 0.15 m, *f* = 0.25 Hz, *U* = 0.25 m/s, and $\alpha_{max}$ = 15° (corresponding St = 0.3). The maximum propulsion efficiency obtained under these conditions was 40.16%, the corresponding mean $C_{x}$ was 0.32, and mean $C_{y}$ was 0.09. The corresponding hydrodynamic characteristic curve is shown in Figure 8.

**Figure 9:**
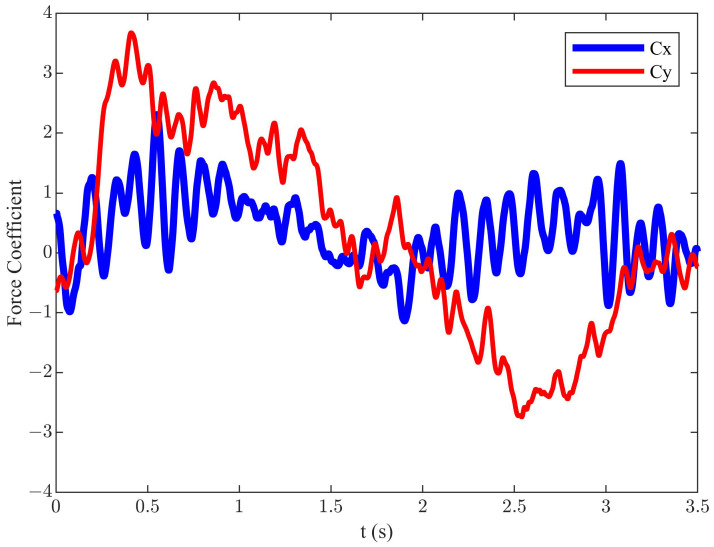
The experiment results of the instantaneous force coefficients of the improved diagonal motion trajectory.

Under the conditions of *h* = 0.15 m, *f* = 0.29 Hz, *U* = 0.25 m/s, β = 125°, $\alpha_{0}$ = −10°, and $\alpha_{max}$ = 45° (corresponding St = 0.348), the maximum propulsion efficiency of the improved diagonal motion trajectory obtained was 22.05%, the corresponding mean $C_{x}$ was 0.37, and mean $C_{y}$ was 0.29. The corresponding hydrodynamic characteristic curve is shown in Figure 9.

It can be observed from Table 2 that the experimental prototype is not as efficient as the numerical simulation. The objective of the simulation was to explore the theoretical maximum propulsion efficiency, while that of the experiment was to explore the actual propulsion efficiency that can be achieved by the flapping-wing prototype under the constraints of the servo drive angular velocity and the skin’s flexible deformation. Due to the limitations of the motion trajectory in engineering implementation, the actual propulsion efficiency was lower than the theoretical value. The actual experimental process cannot achieve the working conditions corresponding to the theoretical maximum propulsion efficiency in the simulation (such as the flapping frequency). At the same time, there is still room for further improvement and optimization of the subsequent flapping-wing prototype vehicle. However, the Strouhal numbers corresponding to the maximum propulsion efficiency obtained under numerical simulation and experimental conditions were both in the range of 0.2 to 0.4, which is also consistent with the Strouhal numbers under the effective propulsion of birds and fish in nature [32].

## 3. Development and Experiment

### 3.1. Design and Manufacture of Prototype Vehicle

**Figure 10:**
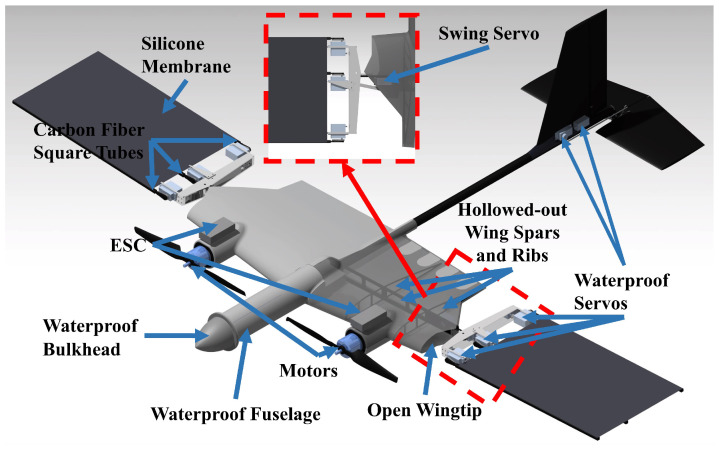
Prototype of the novel aerial-aquatic unmanned vehicle.

For an aerial-aquatic unmanned vehicle, it needs to be able to fly in the air and cruise underwater. Therefore, our solution is based on the design of a fixed-wing aircraft for aerial flight, to which was added a flapping propulsion mechanism for underwater operation. For the flapping propulsion mechanism, we adopted bionic principles, requiring it to achieve symmetric and diagonal flapping motion trajectories. Meanwhile, considering the compatibility between air and underwater environments, we not only needed to waterproof the vehicle but also consider the balance of buoyancy and gravity. Since the aerial-aquatic unmanned vehicle needs to fly in the air, it cannot be too heavy, so we considered suppressing underwater buoyancy. Based on this idea, we developed the prototype shown in Figure 10.

The prototype vehicle is configured as an aft-tail, conventional layout with a wingspan of 2 m, a length of 1.54 m, and a weight of 4.82 kg. The inner section features a fixed-wing design to install propellers and provide lift for aerial flight, while the outer section features a flapping-wing design to ensure underwater propulsion. The wing spars and ribs are constructed from hollowed-out carbon fiber to facilitate rapid, passive flooding and draining. The inclusion of open inner wing segment tips and trailing edge openings (8 drainage holes, each measuring 70 mm in height and 40 mm in width) enables water to readily exit or enter the wing during flooding and draining. The prototype vehicle utilizes an all-movable horizontal tail, aiming to enhance pitch control capabilities in water, thereby facilitating improved control over the pitch attitude of the prototype vehicle during the water egress phase.

The half span of the outer wing segment is 0.6 m, incorporating a rectangular wing design. The flapping-wing mechanism employs a direct drive scheme for the waterproof servos. This design enables control over the vertical flapping of the three fins through three separate waterproof servos, while one waterproof servo governs the swinging motion of the flapping wing around the inner wing. The flapping propulsion mechanism utilizes three 8 mm × 4 mm carbon-fiber square tubes as fin rays, with a 1mm silicone membrane covering the flapping wings. These fin rays are directly actuated by three waterproof servos with a torque rating of 70 kg·cm. These units are mounted on an aluminum alloy frame. The swing servo is mounted on the wing spar of the wingtip of the inner wing section. When the prototype vehicle is conducting aerial flight, the fins located at the leading edge and the middle of the wing are fixed, while the fins located at the trailing edge of the wing can be deflected in the opposite direction to act as an aileron by servo control.

To counteract propeller torque during egress from water, we installed a pair of propeller propulsion systems with reverse rotation on each side of the inner wing segments. We have chosen the SUNNYSKY X4120-7 motor (SUNNYSKY Inc., Zhongshan, China) as the power source, which can be controlled for both positive and negative rotation through the electronic speed controller. It was equipped with the EOLO 15 × 8 propeller, enabling the prototype vehicle to achieve a maximum thrust-to-weight ratio of nearly 2. The motor was regulated by a waterproof electronic speed controller, further fortified with waterproof treatment using Kafuter K-704 Silicone Adhesive (Guangdong Hengda New Material Technology Co., Ltd., Huizhou, China).

As the only part of the prototype vehicle that is waterproof, the fuselage is used to accommodate electronic components such as payloads, the navigation and control computer, the motor power battery, and the servo power battery. The forward bulkhead is connected to the fuselage with bolts and sealed with double O-rings at the connection to prevent water from entering the cabin.

When the prototype vehicle is flying in the air, the flapping-wing mechanism is controlled by the servos to fix the fins, which is similar to a fixed-wing aircraft. Since the prototype vehicle primarily flies at low altitudes and speeds, the Spalart–Allmaras turbulence model was used to conduct aerodynamic simulations. A cruising speed of 15 m/s was set, and the longitudinal aerodynamic numerical simulation was examined in the range of the angles of attack from −6° to 16°. The results showed that the maximum lift-to-drag ratio of this prototype vehicle was 10.34 at 4° angle of attack.

**Figure 11:**
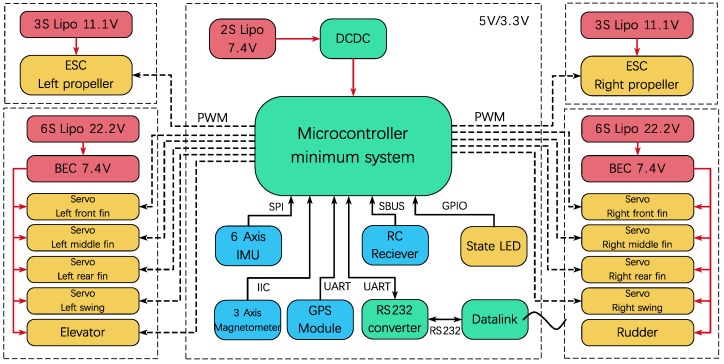
Electronic system block diagram.

**Figure 12:**
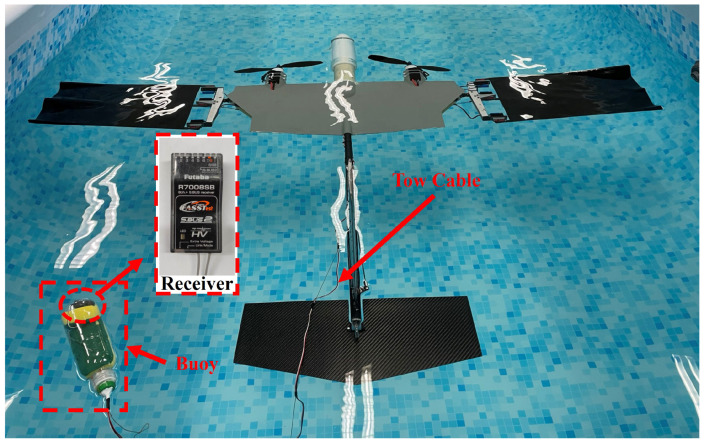
An image of the buoy floating on the water surface to ensure wireless communication.

The prototype vehicle’s electronic and power systems are regulated by a navigation and control computer based on the STM32, along with various onboard sensors. The schematic block diagram of the electronic system of the prototype is shown in Figure 11. The microcontroller minimum system includes a microcontroller, a power supply circuit, a clock circuit, a reset circuit, and a debugging interface. The remote control receiver was installed in the buoy. When the prototype is cruising underwater, the receiver and buoy float on the water surface to ensure normal wireless communication between the receiver and the remote controller. When the receiver receives the wireless commands, it uses the receiver’s SBUS interface to send the command to the navigation and control computer through the tow cable between the buoy and the prototype, as shown in Figure 12.

**Figure 13:**
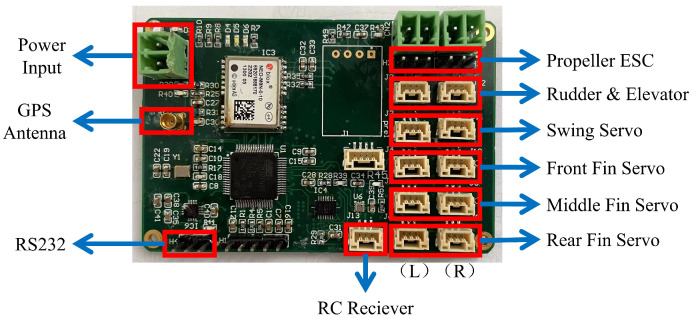
Navigation and control computer.

During aerial flight, the prototype is remotely controlled by the remote controller. However, for underwater operation, where direct observation of the prototype’s motion is not possible, command-based automatic control is used. Commands are sent to the prototype via the remote controller. Once the wireless command signal is received by the remote control receiver, the navigation and control computer analyzes and distributes the commands to each execution component, automatically completing the pre-set actions. This command-based automatic control includes two parts: one is the control of the flapping wing propulsion, and the other is the attitude control of the entire aircraft. Different flapping modes can be switched through remote control commands to achieve different flapping trajectories. Some pre-set flapping modes include the maximum velocity symmetric motion trajectory flapping mode and the maximum velocity diagonal motion trajectory flapping mode. The maximum velocity symmetric motion trajectory flapping mode uses the maximum achievable flapping amplitude of 0.2 m and the maximum achievable frequency of 0.4 Hz. The maximum velocity diagonal motion trajectory flapping mode uses the maximum achievable flapping amplitude of 0.2 m, the maximum achievable frequency of 0.4 Hz, and a stroke angle of 130°. A water egress mode has also been pre-set in the control software. In this mode, the flapping motion is commanded at the maximum velocity diagonal motion trajectory flapping mode to generate the maximum thrust, and the prototype’s pitch angle is automatically maintained at 75°. When reaching the water surface, the propeller is activated and operates at maximum power to pull the prototype out of the water. After reaching a safe height of more than 10 m above the water surface, it switches to remote-control mode for remote-controlled flight. In addition, the prototype’s actions during the landing on the water surface are also preset. When the prototype is remotely controlled to approach the water surface, it switches to deep stall landing mode by the command transmitted via the remote controller. The navigation and control computer controls the deflection of the all-movable horizontal tail to the angle of 20° upward deflection, leading the prototype vehicle to stall and gently descend onto the water surface. All electrical functional modules of the prototype vehicle are consolidated onto a single-board PCB to minimize weight and reduce the risk of interconnect failures, as illustrated in Figure 13. The navigation and control computer measures 7.5 cm × 5 cm × 1 cm, with a weight of 21.8 g.

### 3.2. Experiment and Result

Before the outdoor test, the waterproof performance, flooding and draining, symmetric motion trajectory flapping and diagonal motion trajectory flapping mode, and emergency water exit safety protection mode were tested in the internal field. In the outdoor environment, the egress and ingress test, along with the turning test in the water, were initially conducted to verify the functionalities and effectiveness of the underwater control capability.

The outdoor experiment of the prototype vehicle was conducted in a breezy and open water environment. The experiment was divided into two parts: the underwater cruising speed test and the transmedium transition test. Firstly, an underwater cruise speed test of the prototype vehicle was performed. The Pixhawk4 autopilot (Holybro Inc., Shenzhen, China), equipped with a GPS module for collecting underwater speed, and battery were encapsulated in a waterproof and sealed container and securely attached to the buoy. While underwater cruising, these components remain above the water’s surface and synchronized their movements with the prototype vehicle. The data transmission modules of the host computer and ground station software were utilized for collecting and analyzing prototype vehicle speed data. In the speed test, the working conditions of the prototype vehicle were set as follows: (a) in the symmetric motion trajectory flapping mode, the flapping amplitude of the flapping wings was 0.2 m, and the flapping frequency was 0.4 Hz; (b) in the diagonal motion trajectory flapping mode, the flapping amplitude of the flapping wings was 0.2 m, the flapping frequency was 0.4 Hz, and the stroke angle was 130°.

**Figure 14:**
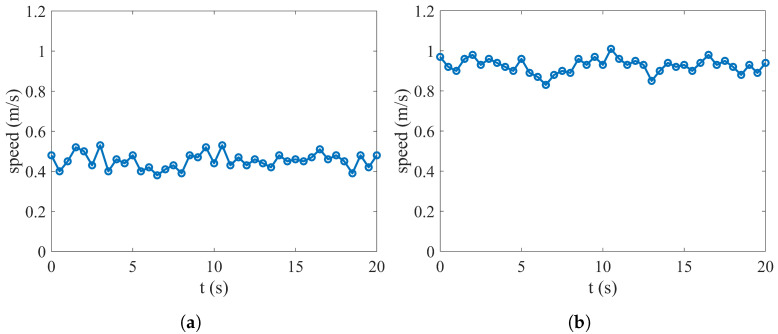
Underwater speed measurement test. (**a**) Symmetric flapping speed. (**b**) Diagonal flapping speed.

The ground station received the speed information that was returned, as shown in Figure 14. In the symmetric motion trajectory flapping mode, the prototype vehicle’s average underwater speed was 0.47 m/s, whereas, in diagonal motion trajectory flapping mode, its average underwater speed was 0.92 m/s.

**Figure 15:**
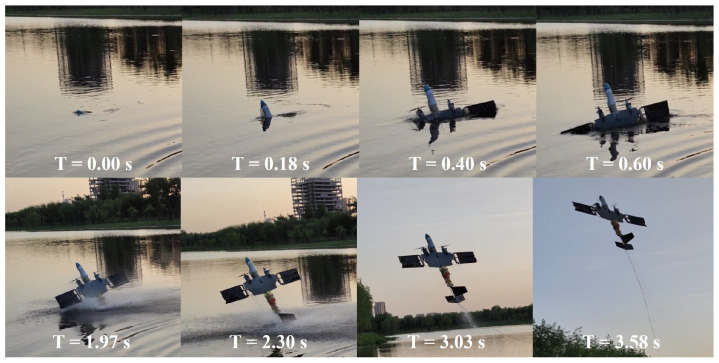
The water-to-air transmedium transition capability test.

In the transmedium transition test, the prototype vehicle was maneuvered to flap on the water’s surface and cruised to the initial position, after which it was ordered to deflect the all-movable horizontal tail downward to create a pitchdown moment and use the flapping wing for propulsion to submerge to a depth of five meters to execute underwater cruising. Subsequently, the prototype vehicle was transitioned to the maximum velocity diagonal motion trajectory flapping mode. Following the stabilization of the underwater propulsion velocity, the mode was switched to the water egress mode. Then, the navigation and control computer autonomously actuated the all-movable horizontal tail to sustain the prototype vehicle at a pitch angle of 75° as it climbed from underwater to the surface. The moment the propeller emerges from the water surface, the control motor propelled the propeller to its maximum thrust. Using the thrust generated by the propellers, the prototype vehicle was elevated from its submerged state and accomplished the transition from underwater submersion to aerial flight, as shown in Figure 15. When the prototype vehicle climbed to a safe altitude of 10 m with a water egress pitch angle, the all-movable horizontal tail was controlled to transition the prototype vehicle from water egress mode to remote control mode and subsequently be controlled by the remote controller. The maximum flight speed in the air reached 27.4 m/s.

**Figure 16:**
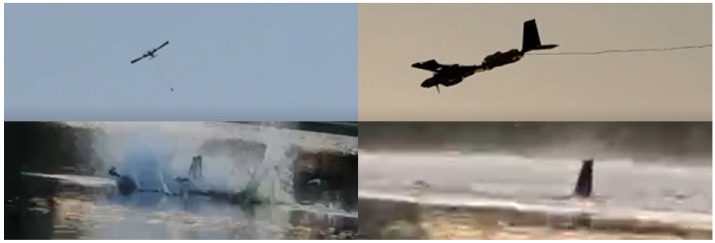
The air-to-water transmedium transition capability test.

Upon completion of the aerial flight, the prototype vehicle was manipulated to descend during gliding. At a distance of 2 to 3 m above the water’s surface, the all-movable horizontal tail was adjusted to an entry angle of 20° upward deflection, controlling the pitch angle to initiate a deep stall landing mode. As the pitch angle was increased, the flight velocity gradually decreased, leading the prototype vehicle to stall and gently descend onto the water surface, thereby accomplishing the transition of the prototype vehicle into the aquatic environment, as illustrated in Figure 16.

## 4. Conclusions

Aerial-aquatic unmanned vehicles represent a synthesis of unmanned aerial vehicles and unmanned underwater vehicles, possessing propulsive capabilities and maneuverability across distinct mediums. To achieve both aerial flight and underwater propulsion, we have developed a prototype vehicle that integrates fixed-wing configuration and flapping-wing configuration. In order to improve the low propulsion efficiency of the classic diagonal trajectory, we proposed an improved diagonal trajectory based on joint optimization of motion trajectory and angle of attack curve. Through simulation result comparison, the efficiency of the improved diagonal trajectory has a 10.1% increase compared to the classical diagonal motion trajectory. In addition, we have designed a flexible flapping wing prototype and conducted water tank experiments on the improved diagonal trajectory, with good flapping efficiency achieved. To verify the performance of the aerial-aquatic unmanned vehicle, we designed and manufactured a prototype vehicle and then conducted the underwater cruising speed test and the transmedium transition test in a natural environment. The results of outdoor experiments proved that the designed aerial-aquatic unmanned vehicle has good transmedium transition capabilities and underwater propulsion performance, for which its average underwater speed is 0.92 m/s.

However, the flexible flapping wing was limited by the angular velocity of the currently employed servos and the restricted range of motion of the elastic skin, resulting in the efficiency not reaching the ideal state. Subsequent efforts will focus on further optimizing and refining the flapping mechanism to enable flapping in both water and air, concurrently achieving full autonomy for the prototype vehicle in transmedium.

## Figures and Tables

**Table 1:** Classical motion trajectory.

Trajectory	Classical Symmetric Motion	Classical Diagonal Motion
Y direction	(1) y(t)=h cos (2πft)	
X direction	(2) x(t)=U·t	(3) $x\left(t\right)=U\cdot t+\frac{y(t)}{tan\;(\beta )}$
Angle of attack	(4) $\alpha \left(t\right)=\alpha_{max}\;sin\;\left(2\pi ft\right)$	(5) $$\alpha \left(t\right)=\left\{\begin{array}{cc} \alpha_{max}\left(0.5-0.5\;cos\;\left(4\pi ft\right)\right) & t<T/2\\ 0 & t⩾T/2 \end{array}\right.$$

**Table 2:** Comparison of results.

Trajectory	Classical Symmetric Motion	Improved Diagonal Motion
Simulation Conditions	*f* = 0.55 Hz, *h* = 0.055 m, *U* = 0.2 m/s, $\alpha_{max}$ = 17° (corresponding St = 0.3025)	*f* = 0.55 Hz, *h* = 0.055 m, *U* = 0.2 m/s, $\alpha_{max}$ = 40°, $\alpha_{0}$ = 5°, β = 144° (corresponding St = 0.3025)
Efficiency	57.16%	34.4%
Experimental conditions	*f* = 0.25 Hz, *h* = 0.15 m, *U* = 0.25 m/s, $\alpha_{max}$ = 15° (corresponding St = 0.3)	*f* = 0.29 Hz, *h* = 0.15 m, *U* = 0.25 m/s, $\alpha_{max}$ = 45°, $\alpha_{0}$ = −10°, β = 125° (corresponding St = 0.348)
Efficiency	40.16%	22.05%

## Data Availability

The original contributions presented in the study are included in the article, further inquiries can be directed to the corresponding author.
